# The defect of SFRP2 modulates an influx of extracellular calcium in B lymphocytes

**DOI:** 10.1186/1756-0500-7-780

**Published:** 2014-11-04

**Authors:** Yuichi Tokuda, Masami Tanaka, Tomohito Yagi, Kei Tashiro

**Affiliations:** Department of Genomic Medical Sciences, Kyoto Prefectural University of Medicine, 465 kajii-cho, Kawaramachi-Hirokoji, Kamigyo-ku, Kyoto, 602-8566 Japan

**Keywords:** SFRP2, PLCγ2, Calcium influx, B cell receptor signaling

## Abstract

**Background:**

In the Wnt pathway, the secreted frizzled-related protein 2 (SFRP2) is thought to act as one of the several competitive inhibitors of Wnt. However, the precise role of SFRP2 is still poorly understood especially in B lymphocytes. Here, we investigated the function of SFRP2, comparing the SFRP2 defective as well as normal B lymphocytes in mice.

**Results:**

We demonstrated that calcium influx from extracellular to intracellular space in splenic B cells was clearly affected by the defect of SFRP2. In addition, the phosphorylation of phospholipase Cγ2 was observed to be reduced in SFRP2 defective splenic B cells with B cell receptor stimulation.

**Conclusions:**

SFRP2 is suggested to modulate the influx from extracellular calcium in the B cell receptor signaling pathway.

**Electronic supplementary material:**

The online version of this article (doi:10.1186/1756-0500-7-780) contains supplementary material, which is available to authorized users.

## Background

The Wnt pathway is one of the important signal mechanisms related to cell differentiation in embryogenesis, hematopoiesis, and carcinogenesis [1]. It is mainly divided into three categories as Wnt/β-catenin, Wnt/planar cell polarity, and Wnt/calcium pathway [2–5]. In particular, the Wnt/β-catenin pathway, which also termed as “canonical pathway”, has been investigated extensively and well understood, comparing to other pathways termed as “noncanonical pathway” [6, 7].

The Wnt protein is one of the extracellular ligands binding to the family of Frizzled receptors associated with several receptor-related proteins. Also, the Wnt pathways are regulated with activators or inhibitors [8]. Especially, the secreted frizzled-related protein 2 (SFRP2) (also known as SDF5 [9]) is a competitive inhibitor to act as antagonist of the Wnt pathway [10, 11].

During embryogenesis, where Wnt signaling is involved, the defect of *Sfrp2* causes brachysyndactyly in mice [12]. Our previous research also showed that the dysfunction of SFRP2 protein yields a phenotype of preaxial synpolydactyly and syndactyly [13]. Moreover, SFRP2 has reported to be hypermethylated in the prostate cancer [14], gastric cancer [15], and colorectal cancer [16], and to suppress bone formation in multiple myeloma cells [17]. On the other hand, the Wnt is known to maintain hematopoietic stem cells (HSCs) in the bone marrow (BM) niche under the both canonical [18] and noncanonical pathways [6], and various Wnt antagonists such as SFRP2 are suggested to play a role in the regulation of HSCs. In the Wnt pathways of hematopoiesis, SFRP2 as secreted protein is suggested to inhibit the Wnt pathway and maintain the quiescent of HSCs in mice [19]. SFRP2 is also known to be expressed in osteoblasts in BM and related to the proliferation of HSCs [20]. However, the function of SFRP2 on immune system is still unclear, especially in the calcium signaling of B lymphocytes.

Here, we demonstrated that SFRP2 modulates the calcium signal transduction associated with activation cascade in downstream of B cell receptor (BCR) signaling pathway.

## Methods

### Mice

Mice of wild-type (*Sfrp2*^*+/+*^) C57BL/6 and of *Sfrp2-*defective strains (*Sfrp2*^-/-^) were bred under the specific pathogen-free (SPF) conditions as described in our previous study [13]. In this study, all mice were examined at 10-12 weeks of age. Reproducibility of data was confirmed by repeating each experiment at least more than three pairs of *Sfrp2*^*+/+*^ and *Sfrp2*^-/-^. All procedures in mouse experiments followed the guidelines and were approved by the Kyoto Prefectural University of Medicine Animal Care and Use Committee.

### Cell preparation

The cell suspensions were obtained from the BM and spleen samples. After the elimination of red blood cells, the cells in the BM or spleen were suspended in phosphate buffered saline (PBS) with 3% fetal bovine serum. For the western blotting, the splenic B cells were purified by negative isolation using Dynabeads® Mouse CD43 (Untouched™ B Cells) (Invitrogen, Carlsbad, CA, USA) according to the manufacturer’s protocol.

### Cell differentiation analysis

BM cells were stained by the monoclonal antibodies against the surface markers as follows: FITC-conjugated anti-IgM (II/41), anti-CD43 (S7) and APC-conjugated anti-CD45R/B220 (RA3-6B2; anti-B220). Splenocytes were similarly stained by the monoclonal antibodies as follows: FITC-conjugated anti-CD21/CD35 (7G6), PE-Cy7-conjugated anti-IgM (R6–60.2), anti-B220 antibody (BD Pharmingen, San Diego, CA, USA) and PE-conjugated anti-CD23 (B3B4) antibody (eBioscience, San Diego, CA, USA). All flow cytometry (FACS) experiments were performed by BD FACS Canto II and BD FACSDiva software version 6.1.3 (BD Biosciences, San Jose, CA, USA) according to the manufacturer’s protocol. The analysis was performed using the FlowJo software (Tree Star, San Carlos, CA, USA).

### Calcium influx analysis

Cell suspensions of splenocytes were incubated at 37°C for 45 min with Fluo 4-AM (Fluo4; Dojindo, Kumamoto, Japan) and Fura Red™ AM (Fura Red; Invitrogen), which final concentrations were 3 μM and 6 μM, respectively. After stained with anti-B220, the cells were resuspended in calcium-free Hank’s balanced salt solution (HBSS/Ca^−^). Intracellular calcium levels were assessed by the ratio of the intensities of Fluo4/Fura Red [21], which the ratios were averaged for every 10 sec. In the experiments, anti-mouse IgM F(ab’)_2_ fragments (anti-IgM; final concentration 10 μg/ml in HBSS/Ca^−^, Jackson Immunoresearch, West Grove, PA, USA) and ethylene glycol bis(2-aminoethyl)-N,N,N’,N’-tetraacetic acid (EGTA; final concentration 0.5 mM in HBSS/Ca^−^), and calcium (Ca; CaCl_2_ final concentration 1.26 mM in HBSS/Ca^−^) were applied. These experiments were replicated at least three times.

### Endoplasmic reticulum (ER) analysis

The splenocytes were incubated at 37°C in 5% CO_2_ for 45 min in PBS (Mg^+^, Ca^+^) and 1 μM ER-Tracker™ Green dye (glibenclamide BODIPY® FL, ER-Tracker; Molecular Probes, Invitrogen) to evaluate the ER abundance [22]. After staining with anti-B220 for 10 min, the cells were resuspended in PBS (Mg^+^, Ca^+^) for FACS analysis.

### Reverse Transcription Polymerase Chain Reaction (RT-PCR)

RT-PCR experiments were performed with Multiple Tissue cDNA (MTC) panels of Mouse (Clontech Laboratories, CA, USA) and *Sfrp2*^+/+^ and *Sfrp2*^-/-^ samples, which were from only *Sfrp2*^*+/+*^ and both mouse for SFRP2 and β-catenin tests, respectively. The cDNAs from *Sfrp2*^+/+^ and *Sfrp2*^-/-^ mouse samples were synthesized in 20 μl products with 200 ng total RNA from splenic B and BM cells and Super Script II (Invitrogen) according to the manufacturer’s protocol. In the PCR process, each cDNA in appropriate mixture was amplified with each specific primer pair, and their details were described in Additional files 1 and 2.

### Protein phosphorylation assay by FACS

The phosphorylation assay of proteins was measured by FACS with BD™ Phosflow technology (BD Biosciences) according to the manufacturer’s instructions. The stimulations for splenic B cells were examined by anti-IgM antibody (final concentration 10 μg/ml; Jackson Immunoresearch) or lipopolysaccharide (LPS, final concentration 20 μg/ml; Sigma-Aldrich, San Francisco, CA, USA) in time course of 0, 5, 10, and 15 min. In the case of IgM stimulation, the antibody set of Alexa Fluor® 488 Mouse ERK1/2 (pT202/pY204) (Erk1/2) and PE Mouse anti-Syk (pY348) (Syk) was applied to detect the phosphorylated proteins. In the case of LPS stimulation, Erk1/2 and PE Mouse p38 MAPK (pT180/pY182) (P38) (BD™ Phosflow, BD Biosciences) were examined. These antibodies with anti-B220 were stained for splenic B cells for 30 min in Phosflow experiment process.

### Western blotting

The purified splenic B cells were stimulated by anti-IgM (10 μg/ml, Jackson) in HBSS with calcium. The samples were evaluated by antibodies from Antibody Sampler Kits (Cell Signaling Technology, Inc. (CST), Danvers, MA, USA) as follows: anti-phospho-Syk (Tyr525/526), anti-Syk, anti-phospho-Lyn (Tyr507), anti-Lyn, anti-phospho-Btk (Tyr223), anti-Btk, anti-phospho-CD19 (Tyr531), and anti-CD19 from B Cell Signaling Antibody Sampler Kit; anti-phospho-PLCγ2 (Tyr1217), anti-phospho-PLCγ2 (Tyr759), and anti-PLCγ2 antibody from PLCγ Antibody Sampler Kit; anti-phospho-SAPK/JNK (Thr183/Tyr185), and anti-phospho-ATF-2 (Thr71) from Phospho-SAPK/JNK Pathway Antibody Sampler Kit. Moreover, anti-NFAT1, anti-NFAT2, and β-actin antibody (CST) were also applied. These antibodies were detected with anti-rabbit IgG-HRP (CST) as secondary antibody. The signals were detected with the ECL Prime or ECL Plus Western Blotting Detection System (GE Healthcare UK Ltd., Buckinghamshire, UK) according to the manufacturer’s protocol. In addition, the Can Get Signal Immunoreaction Enhancer Solution (Toyobo Co., Ltd, Osaka, Japan) was applied if necessary. The results of western blots were analyzed by ImageJ software (http://imagej.nih.gov/ij/index.html).

### Statistical analysis

In order to prepare the FACS data for statistical analysis, Office Excel and Visual C++ (Microsoft, Redmond, Washington, USA) were used. We employed R software (http://www.R-project.org/) to perform the statistical analysis including *t*–test in each FACS data and draw the graphs. In the histograms, error bars indicate standard deviation with mean. In addition, the R package of “exactRankTests” was used for Wilcoxon tests.

## Results

### Cell differentiation

**Figure 1:**
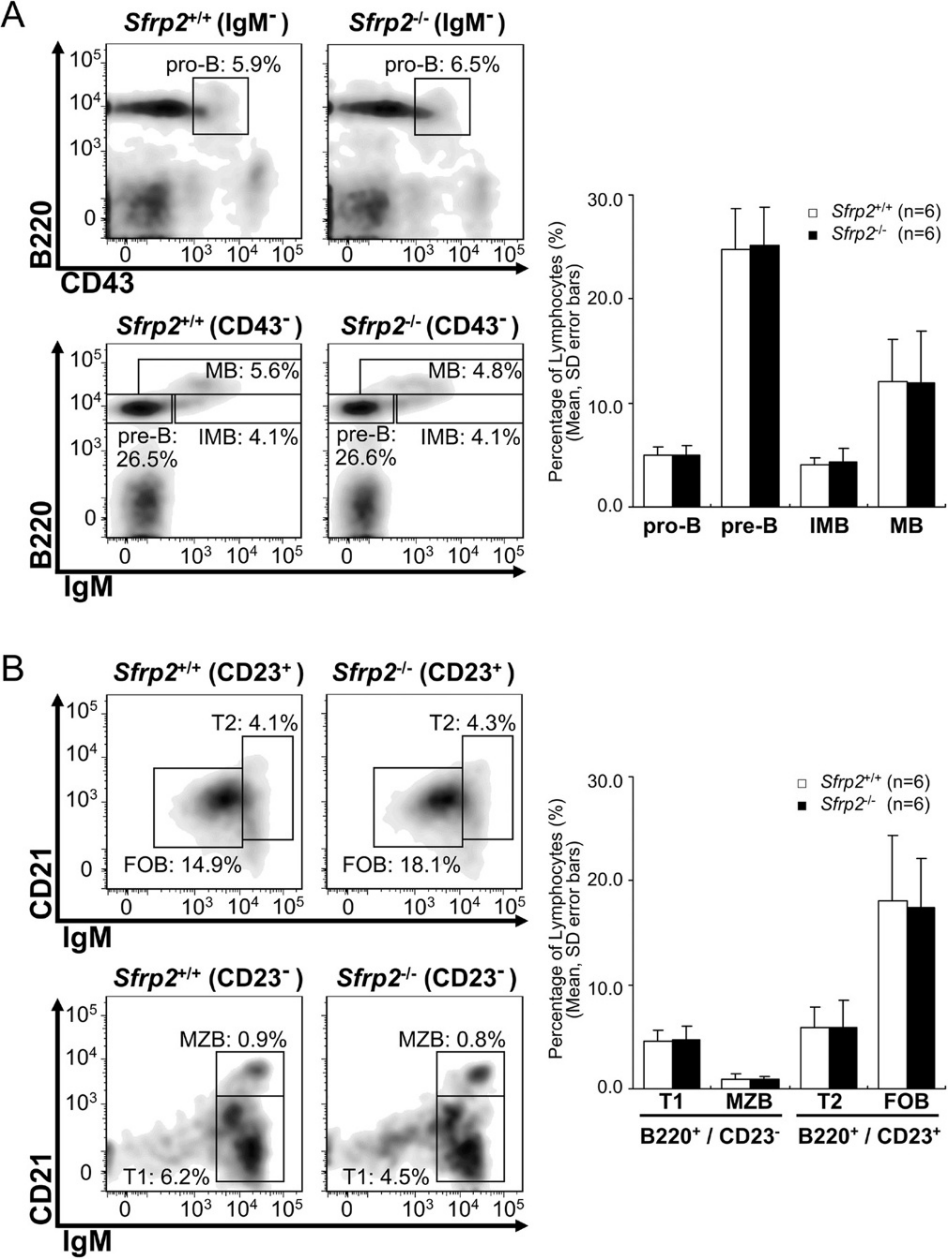
**Comparison of BM and splenic B cell differentiation.** The cells in the BM or spleen were assessed with each surface marker. The representative FACS plots are demonstrated by FlowJo. **(A)** The results of BM are indicated for B cell differentiation stages of pro-B, pre-B, immature B (IMB), and mature B (MB) cells. The histograms indicate the means and SD of the 6 littermates with same gender pairs for these cell stages. No statistical significant difference between *Sfrp2* ^+/+^ and *Sfrp2* ^-/-^ in these cell stages was observed by Student’s *t*-tests. **(B)** The results of splenic B cells for transitional type 1 (T1), marginal zone B (MZB), transitional type 2 (T2), and follicular B (FOB) cells are indicated. The histograms indicate the means and SD of the 6 gender-matched littermates. In splenic B cells, there was no statistical significant difference between *Sfrp2* ^+/+^ and *Sfrp2* ^-/-^ by Student’s *t*-tests.

In order to evaluate the differences in B cell differentiation, BM and splenic B cells obtained from *Sfrp2*^+/+^ and *Sfrp2*^-/-^ mice were assessed by FACS analysis. No change in B cell differentiation was observed between *Sfrp2*^+/+^ and *Sfrp2*^-/-^ mice by the statistical analysis (Figure 1).

### Calcium influx

**Figure 2:**
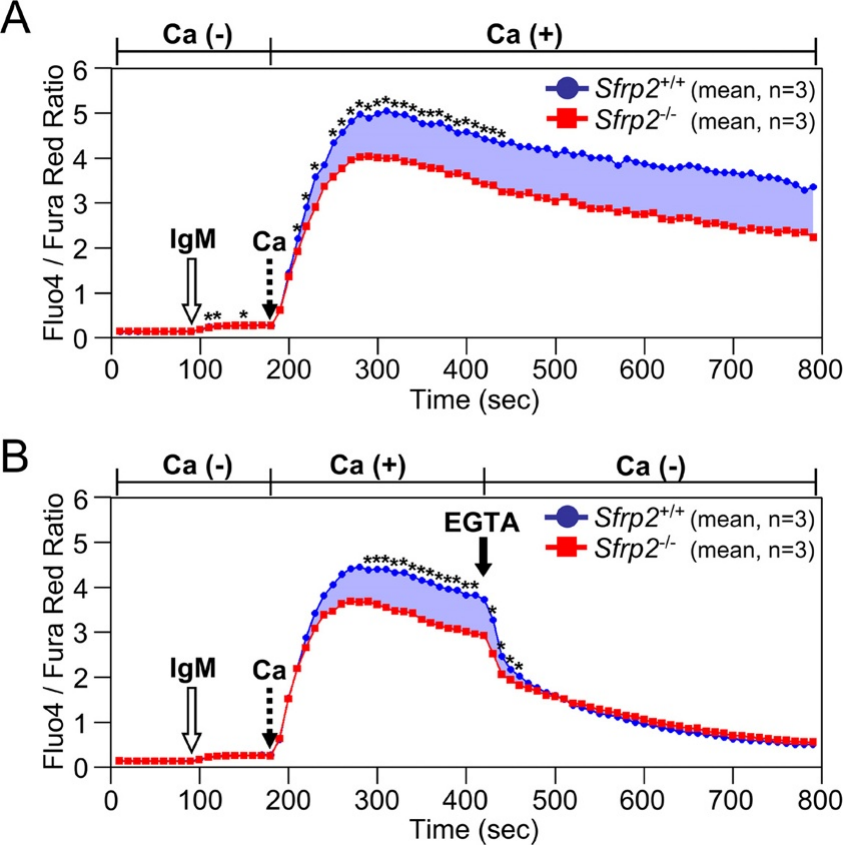
**Calcium influx of splenic B cell.** The calcium influx in splenic B cells was examined by FACS. The *Sfrp2* ^+/+^ and *Sfrp2* ^-/-^ splenic B cells were derived from 3 littermates with same gender pair and assessed after gated with anti-B220. The means of 3 replicates are plotted by the blue circles (*Sfrp2* ^+/+^) and red squares (*Sfrp2* ^-/-^). The black asterisks indicate the statistical significance by paired *t*-test in each time point. Blue shaded regions indicate the differences of intensity ratios between *Sfrp2* ^+/+^ and *Sfrp2* ^-/-^ splenic B cells. **(A)** After 1.5 min acquisition of signals, the cells were stimulated with anti-IgM (IgM; open arrow). Furthermore, the cells were treated with calcium at 3 min (dotted arrow). **(B)** Following the similar process, the cells were then incubated with EGTA at 7 min (filled arrow).

The differences of calcium signal transduction between *Sfrp2*^+/+^ and *Sfrp2*^-/-^ splenic B cells were evaluated by FACS. No significant difference of intracellular calcium levels was observed with anti-IgM stimulation under the calcium free condition (open arrows in Figure 2). After the addition of extracellular calcium (dotted arrows in Figure 2), intracellular calcium levels rapidly increased because of the influx of extracellular calcium after anti-IgM stimulation. Once reaching the peak, intracellular calcium levels were then gradually decreased with the statistical significant difference between *Sfrp2*^+/+^ and *Sfrp2*^-/-^ splenic B cells (Figure 2A). However, the difference of intracellular calcium levels between *Sfrp2*^+/+^ and *Sfrp2*^-/-^ splenic B cells rapidly disappeared after the removal of extracellular calcium by EGTA (filled arrow in Figure 2B).

### ER abundance analysis

**Figure 3:**
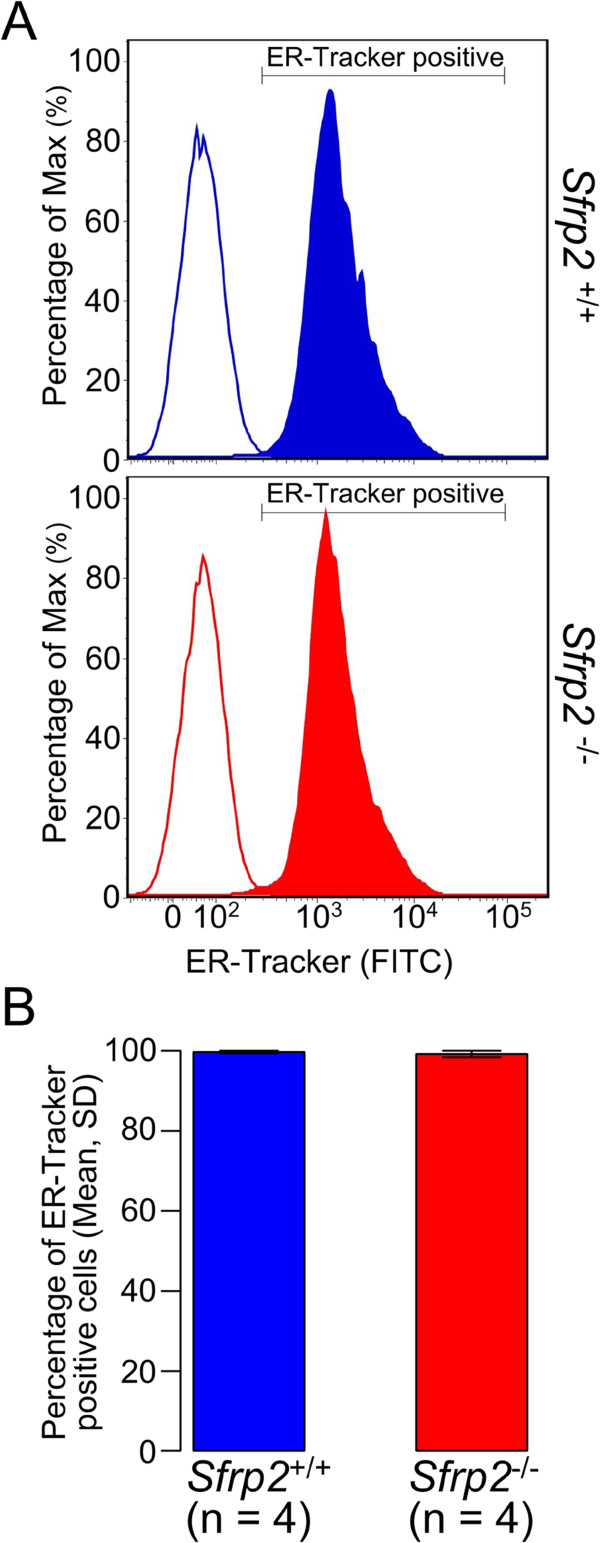
**ER abundance of splenic B cell. (A)** The representative results of ER abundance in splenic B cells gated with anti-B220 are displayed by using FlowJo. Upper (blue) and lower (red) plots are represented as the percentages of the max of the ER-Tracker signals in splenic B cell of *Sfrp2* ^+/+^ and *Sfrp2* ^-/-^, respectively. The filled areas indicate ER-Tracker stained B cells and the line areas indicate the intensity of non-stained samples. **(B)** The histograms indicate the means and SD of the 4 littermate pairs for the percentage of ER-Tracker positive cells. No statistical significant difference in ER abundance between *Sfrp2* ^+/+^ and *Sfrp2* ^-/-^ splenic B cells was observed by Student’s *t*-tests.

ER abundance in splenic B cells was evaluated with ER-Tracker. The results showed that there was no statistical significant difference for ER abundance between the *Sfrp2*^+/+^ and *Sfrp2*^-/-^ splenic B cells (Figure 3).

### SFRP2 and β-catenin expression

The expression of SFRP2 studied by RT-PCR was high in BM, but very low in spleen/splenic B cells (Additional file 1). On the other hand, the expression of β-catenin was clearly noted in spleen similar to other tissues, as well as in *Sfrp2*^+/+^ and *Sfrp2*^-/-^ splenocytes (Additional file 2A and B). However, phosphorylated β-catenin was barely detectable in BM or spleen tissues by Western blotting (Additional file 2C).

### Western blotting analysis on phosphorylated protein

Before western blots analysis, we first tested each phosphorylation of Erk1/2, P38, and Syk in *Sfrp2*^+/+^ and *Sfrp2*^-/-^ splenic B cells with use of FACS (Additional file 3). Results showed that there was no significant phosphorylation difference between *Sfrp2*^+/+^ and *Sfrp2*^-/-^ when stimulated with either IgM or LPS. Therefore, we examined phosphorylation status of the proteins involved in the BCR signaling pathway with use of western blotting for purified splenic B cells. The purity of splenic B cells was about 98.6% in lymphocytes confirmed by FACS analysis (data not shown).

**Figure 4:**
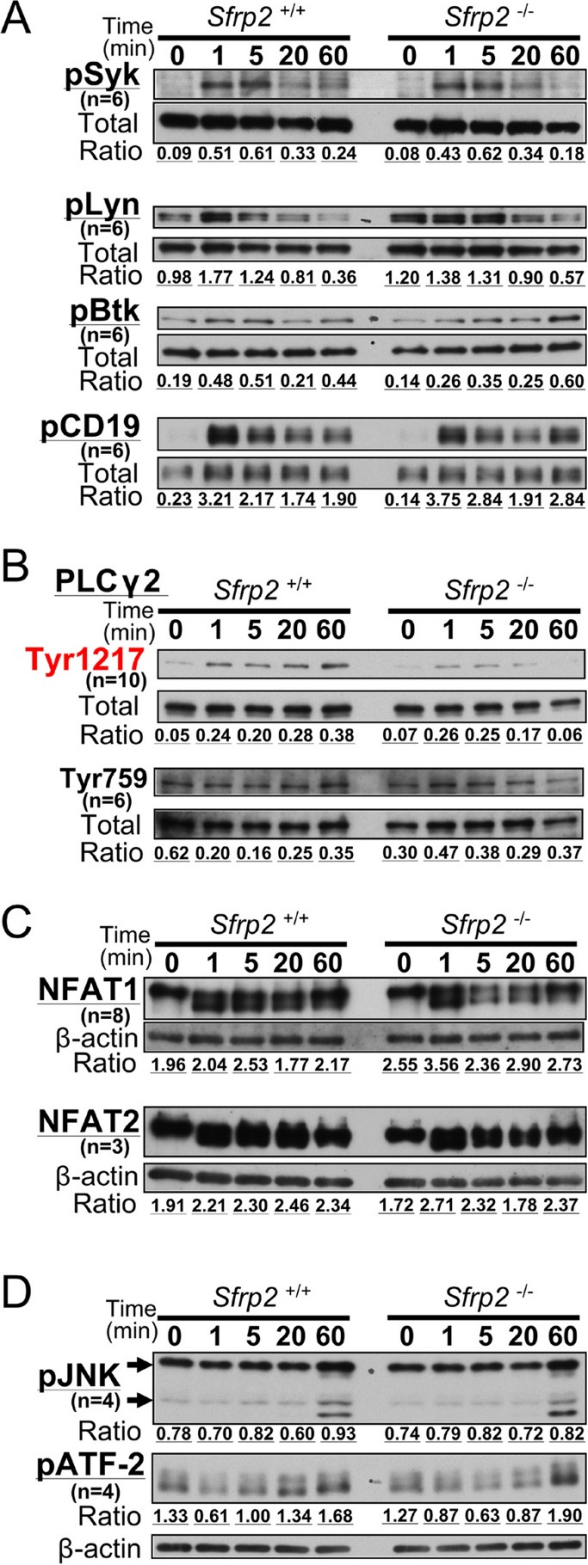
**Western blotting results of PLCγ2 splenic B cell.** The representative results of western blotting were displayed. Splenic B cells were stimulated with anti-IgM. All experiments were replicated and confirmed three times at least. “n” indicates the number of total tested sample for each protein. **(A)** The phosphorylation of Syk (Tyr525/526; pSyk), Lyn (Tyr507; pLyn), Btk (Tyr223; pBtk), and CD19 (Tyr531; pCD19) sites and **(B)** Tyr1217 and Tyr759 phosphorylation of PLCγ2 were demonstrated with “Total” as the controls, which indicate the amount of each applied protein. **(C)** The expressions of NFAT1 and NFAT2 were indicated with β-actin. **(D)** The phosphorylation of SAPK/JNK (Thr183/Tyr185; pJNK) and ATF-2 (Thr71; pATF-2) were indicated with β-actin. Note that there were two bands for JNK in 54 and 46 kDa due to isoforms as noted by arrows. The ratio of expression level of each sample was calculated by using ImageJ.

The phosphorylation of Syk, Lyn, Btk, CD19, and PLCγ2, which is the important downstream effectors of BCR signaling pathway, were assessed comparing with the total amount of each protein. In the western blot analysis, no difference between *Sfrp2*^+/+^ and *Sfrp2*^-/-^ splenic B cells was observed in the time-course experiments after BCR stimulation with anti-IgM (0, 1, 5, 20, and 60 min) (Figure 4A). However, in the Tyr1217 site of PLCγ2, the signals of *Sfrp2*^-/-^ splenic B cells decreased after 5 min stimulation compared with *Sfrp2*^+/+^ samples (Figure 4B). By contrast, no significant difference was observed in the phosphorylation of PLCγ2 at Tyr759 in this study. Accordingly, the defect of *Sfrp2* was considered to affect the phosphorylation of PLCγ2 at Tyr1217 but not Tyr759 in the BCR signaling pathway.

In addition, NFAT1 and NFAT2 were investigated as downstream components of PLCγ2 in the BCR signaling (Figure 4C). Because there was no difference in these proteins between *Sfrp2*^+/+^ and *Sfrp2*^-/-^, the defect of *Sfrp2* was considered not to play a role in the downstream of PLCγ2. Also, in the downstream of calcium signaling cascade related to BCR signaling pathway, no significant difference of phosphorylation in JNK and ATF-2 was found between *Sfrp2*^+/+^ and *Sfrp2*^-/-^ splenic B cells (Figure 4D).

## Discussion

In this study, we investigated B lymphocytes in mice affected by the defect of *Sfrp2*, and this defect did not yield the remarkable influence on the early differentiation of B cells (Figure 1). Therefore, we further examined mature B cells in spleen about the influence of the defect of *Sfrp2* in intracellular signal transduction in detail.

The calcium signaling plays a very critical role in the immune system including B cells [23], and so the calcium influx for splenic B cells with *Sfrp2* defect was selectively examined. We showed that the calcium signal transduction by BCR activation was slightly increased in *Sfrp2*^+/+^ as well as *Sfrp2*^-/-^ splenic B cells under calcium free condition (open arrows in Figure 2). Moreover, no difference of ER abundance was observed between these B cells (Figure 3). Thus, we could conclude that no significant difference was observed in the intracellular calcium store in both *Sfrp2*^+/+^and *Sfrp2*^-/-^ splenic B cells. However, when the calcium was added in the extracellular space (dotted arrows in Figure 2), intracellular calcium levels were rapidly increased in both *Sfrp2*^+/+^ and *Sfrp2*^-/-^ splenic B cells due to the influx of extracellular calcium by the BCR stimulation. This was considered to be attributed to the activation of calcium release-activated calcium channel in the plasma membrane triggered by emptying of ER calcium stores under calcium free condition and the first IgM stimulation (open arrows in Figure 2) [23]. Subsequently, intracellular calcium levels gradually decreased and differed significantly between *Sfrp2*^+/+^ and *Sfrp2*^-/-^ splenic B cells (Figure 2A). By contrast, intracellular calcium levels were rapidly decreased to the same levels in both splenic B cells after EGTA addition (Figure 2B). Therefore, this phenomenon was observed as a result of the difference of the calcium influx from extracellular to intracellular space between *Sfrp2*^+/+^ and *Sfrp2*^-/-^ splenic B cells.

This calcium influx phenomenon is known to be associated with the activation of several proteins involved in the regulation of cell homeostasis. Specifically, protein tyrosine kinases such as Syk and Lyn are initially activated in response to BCR stimulation, which leads to the activation of Btk and CD19. PLCγ2 is then activated by Btk, and cleaves phosphatidylinositol-bisphosphate (PIP_2_) into diacylglycerol and inositol (1, 4, 5)-trisphosphate (IP_3_) by hydrolysis. Subsequently, IP_3_ induced calcium release from intracellular ER calcium stores by binding to the IP_3_ receptor. The catalytic hydrolysis of PIP_2_ is suggested to require the phosphorylation of Tyr759 in PLCγ2 [24, 25]. Moreover, the role of Tyr759 phosphorylation is considered to be different from that of Tyr1217 in PLCγ2 according to the types of cells or stimulations [26]. Our results clearly showed that the defect of *Sfrp2* does not affect the phosphorylation of Syk, Lyn, Btk, and CD19, but reduces the phosphorylation of PLCγ2 at Tyr1217, whereas Tyr759 phosphorylation remained unaffected (Figure 4B). This result may indicate that the *Sfrp2* participates in not pivotally regulating the catalytic hydrolysis of PIP_2_ but modulating the calcium signal transduction.

It was unknown if the effect of these defective *Sfrp2* on PLCγ2 is correlated with other abnormal mechanisms in the canonical and/or non-canonical pathways. First, since SFRP2 is not expressed in the hematopoietic cells, especially in splenic B cells compared to BM cells in *Sfrp2*^+/+^ mice (Additional file 1), exogenous SFRP2 provided from other tissues may contribute to the calcium signaling in the splenic B cell. Moreover, since β-catenin is rarely detectable as protein levels in these splenic B cells (Additional file 2), exogenous SFRP2 may act on the calcium signaling through non-canonical pathway. However, NFAT1, NFAT2, JNK, and ATF-2, which are considered as members of a cascade in downstream of non-canonical signaling pathway, were found not to play a significant role in the *Sfrp2*^-/-^ splenic B cells (Figure 4C and D). Taken together, the dysregulation of calcium signaling in the *Sfrp2*^-/-^ splenic B cells occurs under BCR stimulation and is likely to be correlated with unknown common underlying signal pathway(s) of both BCR and non-canonical signalings.

As previously reported, the expression of SFRP2 was down-regulated by methylation in cancer [14, 15]. Because calcium signaling was reduced by defect of *Sfrp2*, down-regulation of SFRP2 is assumed to impair the calcium signal transduction in each tissue or cell. However the immune dysfunction was not observed in our SFRP2 deficient mice under the SPF condition, it was reported the association between the methylation of SFRP2 and cancer [14–17]. Although further examination is needed, our results might give us the new insights to understand the functions of SFRP2 under the BCR and calcium signal pathway and the mechanisms of several human diseases.

## Conclusions

The defect of *Sfrp2* in mice splenic B cells causes the impairment of calcium influx and the activation of PLCγ2 in the BCR signaling pathway. This phenomenon is speculated to be indirectly related to the activations of Wnt pathways.

## Electronic supplementary material

Additional file 1: **The RT-PCR results for SFRP2.** (PDF 90 KB)

Additional file 2: **The expression analyses for β-catenin.** (PDF 440 KB)

Additional file 3: **The results of the phosphorylation experiments with splenic B cells**. (PDF 98 KB)
