# Supplementary material for: The defect of SFRP2 modulates an influx of extracellular calcium in B lymphocytes
Source: BMC Res Notes. 2014 Nov 4;7:780. doi: 10.1186/1756-0500-7-780 (PMC4242488; doi:10.1186/1756-0500-7-780)
Supplement: Supplementary file 1 — Additional file 1: The RT-PCR results for SFRP2. (PDF 90 KB) [file 13104_2013_3323_MOESM1_ESM.pdf]

Additional File 1: The RT-PCR results for SFRP2.

A

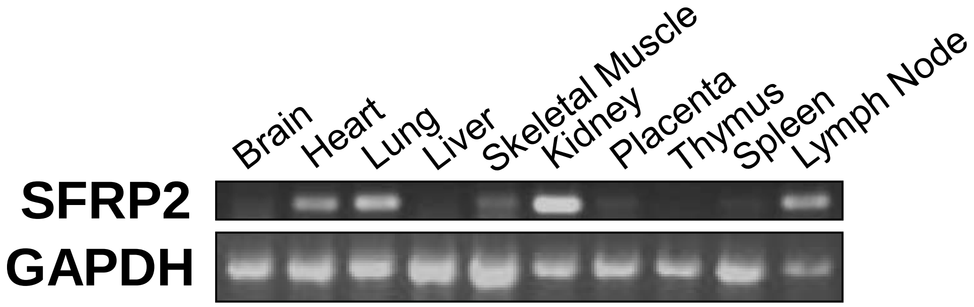

B

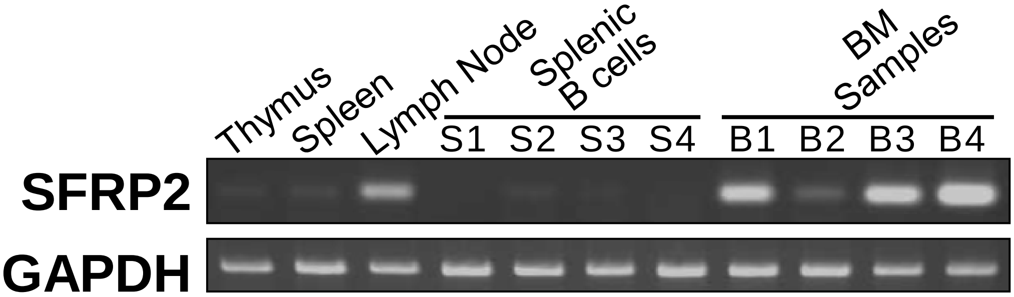

Gene	Primer sequences
**SFRP2**	**F 5'- ATCCTGGAGACAAAGAGCAAGACC -3'**
**(142 bp)**	**R 5'- TGACCAGATACGGAGCGTTGATG -3'**
**GAPDH**	**F 5'- TGAAGGTCGGTGTGAACGGATTTGGC -3'**
**(983 bp)**	**R 5'- CATGTAGGCCATGAGGTCCACCAC -3'**

(A) Each 1 μl cDNA from total 10 tissues of mouse MTC panels was amplified with SFRP2 and GAPDH primers. PCR condition was as follows: initial denaturation at 94˚C for 30s; 30 cycles at 94˚C for 10s, 68˚C for 10s and 72˚C for 1min; and final extension 72˚C for 1min.

(B) Based on the results of (A), each 0.5 μl cDNA from MTC panels (Thymus, Spleen, and Lymph Node) and the *Sfrp2*$^{+/+}$ mouse (Splenic B cells from sample S1 to S4, and BM from sample B1 to B4) was amplified with SFRP2 and GAPDH primers. PCR condition was as follows: initial denaturation at 94˚C for 30s; 5 cycles at 94˚C for 10s, 66˚C for 20s and 72˚C for 45s; 35 touch-down PCR cycles at 94˚C for 10s, 66 to 62.5˚C for 20s and 72˚C for 45s; and final extension 72˚C for 2min.
