# Supplementary material for: The defect of SFRP2 modulates an influx of extracellular calcium in B lymphocytes
Source: BMC Res Notes. 2014 Nov 4;7:780. doi: 10.1186/1756-0500-7-780 (PMC4242488; doi:10.1186/1756-0500-7-780)
Supplement: Supplementary file 2 — Additional file 2: The expression analyses for β-catenin. (PDF 440 KB) [file 13104_2013_3323_MOESM2_ESM.pdf]

Additional File 2: The expression analyses for β-catenin.

A

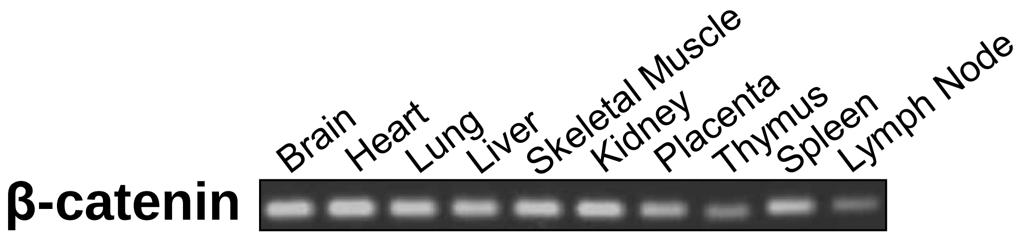

Gene	Primer sequences
β-catenin	F 5'- AGCCGAGATGGCCCAGAAT -3'
(127 bp)	R 5'- AAGGGCAAGGTTTCGAATCAA -3'

B

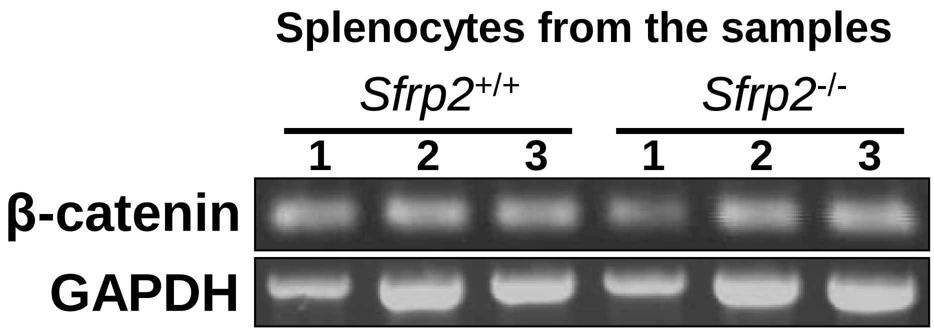

C

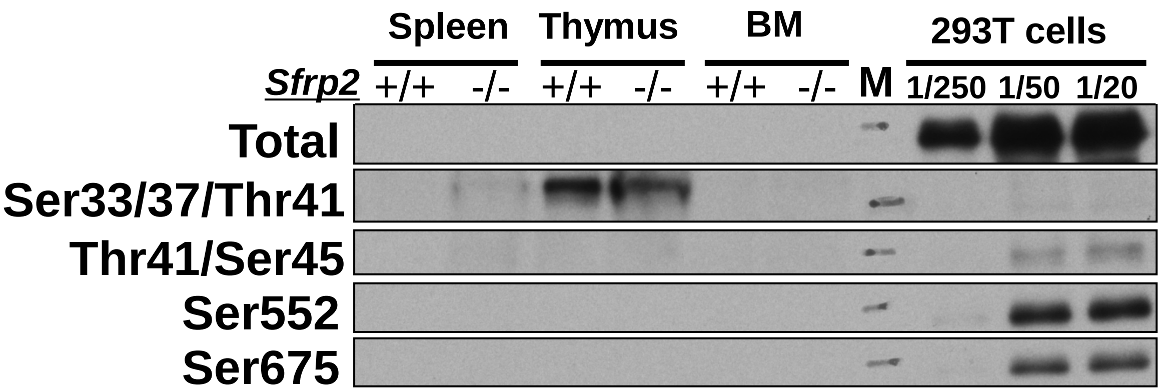

(A) As the same of Additional File 1, each 1 μl cDNA from total 10 tissues of mouse MTC panels was amplified with β-catenin primers. PCR condition was as follows: initial denaturation at 96 ˚C for 1min; 32 cycles at 96˚C for 10s, 62˚C for 10s and 72˚C for 30s.

(B) Based on the results of (A), RT-PCR for β-catenin was also examined with 1 μl cDNA from the mice, which consisted of three littermate pairs (1, 2, and 3) with same gender. Sample from *Sfrp2*$^{+/+}$ and *Sfrp2*$^{-/-}$ are indicated by (+) and (-), respectively. PCR condition was as follows: initial denaturation at 96 ˚C for 1min; 38 cycles at 96˚C for 10s, 62˚C for 10s and 72˚C for 30s.

(C) Western blotting for β-catenin from the mice samples was performed with 293T cells as the positive controls. The samples of spleen, thymus, and BM were from same mice pair of same gender littermates and fixed to $1×10^7$ cells per lane. In the figure, “+/+” and “-/-” mean the cells from *Sfrp2*$^{+/+}$ and *Sfrp2*$^{-/-}$ mouse, respectively. 293T cells were fixed to $5×10^7$ cells and diluted to 1/20, 1/50, and 1/250. For the detection of β-catenin, the samples were evaluated by antibodies as follows: β-Catenin (D10A8), Phospho-β-Catenin (Ser33/37/Thr41), Phospho-β-Catenin (Thr41/Ser45), Phospho-β-Catenin (Ser552) (D8E11), Phospho-β-Catenin (Ser675) (D2F1), from β-Catenin Antibody Sampler Kit (CST). “M” in the figure indicates the size of 100kDa and all molecular weight of β-catenin are 92kDa. Therefore, the band in the spleen or thymus of “Ser33/37/Thr41” is considered not to be β-catenin because of its smaller molecular weight. We repeated this experiment and consideration of conditions in three times at least.
