# Supplementary material for: The defect of SFRP2 modulates an influx of extracellular calcium in B lymphocytes
Source: BMC Res Notes. 2014 Nov 4;7:780. doi: 10.1186/1756-0500-7-780 (PMC4242488; doi:10.1186/1756-0500-7-780)
Supplement: Supplementary file 3 — Additional file 3: The results of the phosphorylation experiments with splenic B cells. (PDF 98 KB) [file 13104_2013_3323_MOESM3_ESM.pdf]

Additional File 3: The results of the phosphorylation experiments with splenic B cells.

A

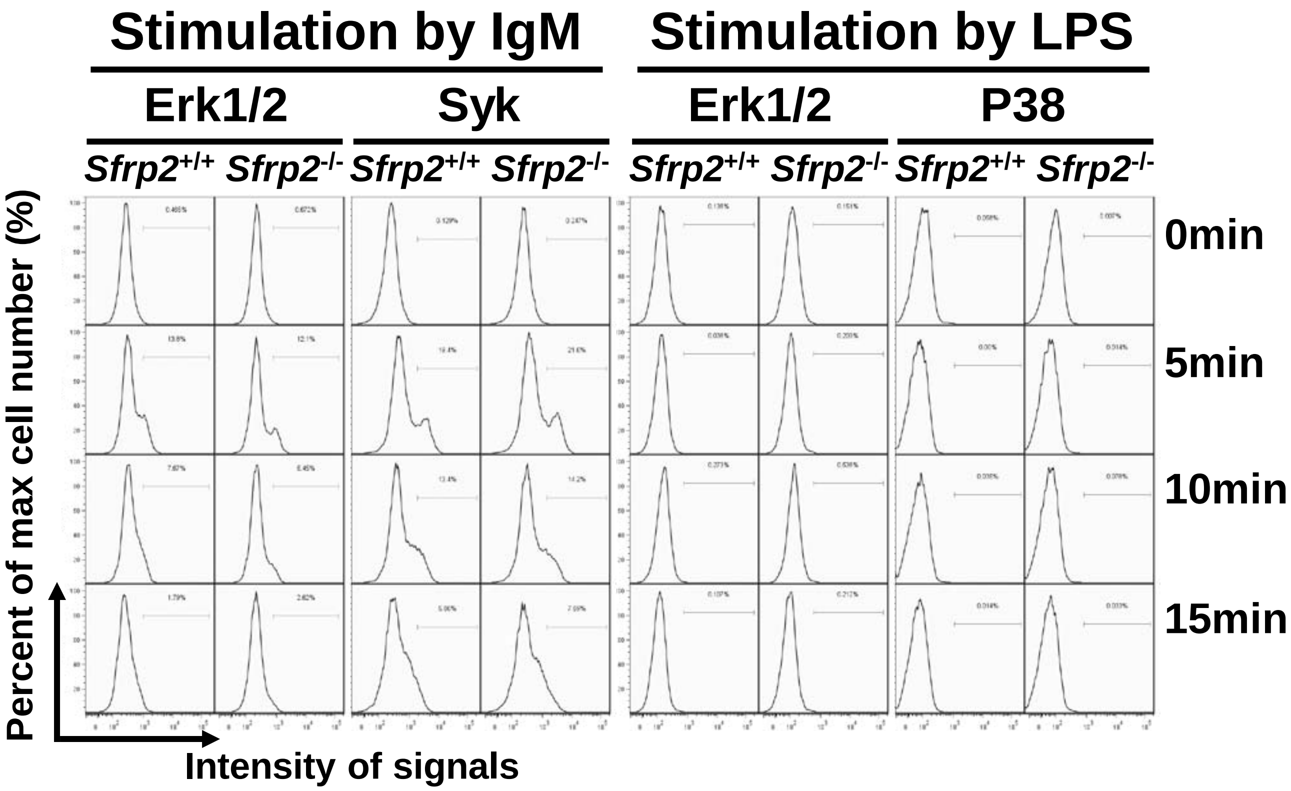

B

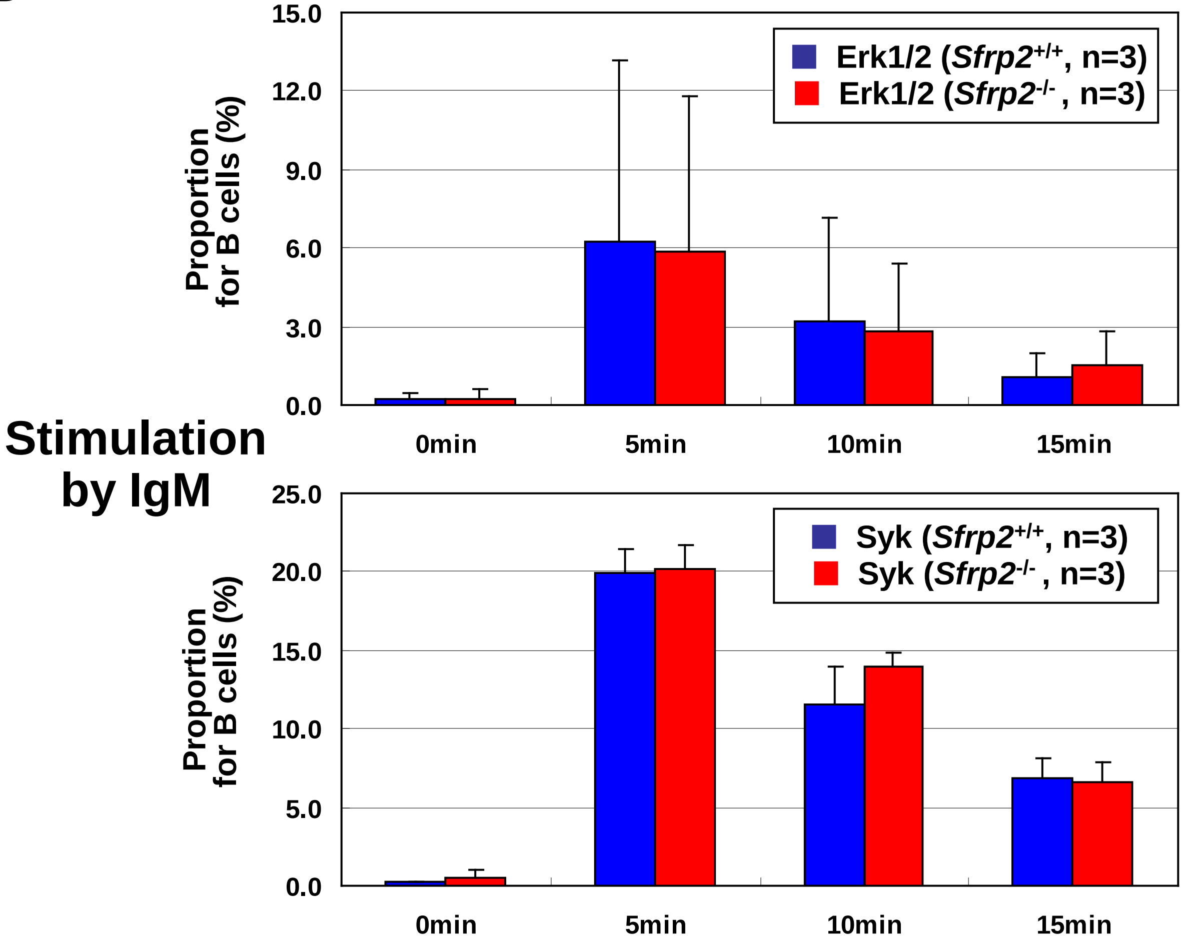

C

Stimulation by LPS

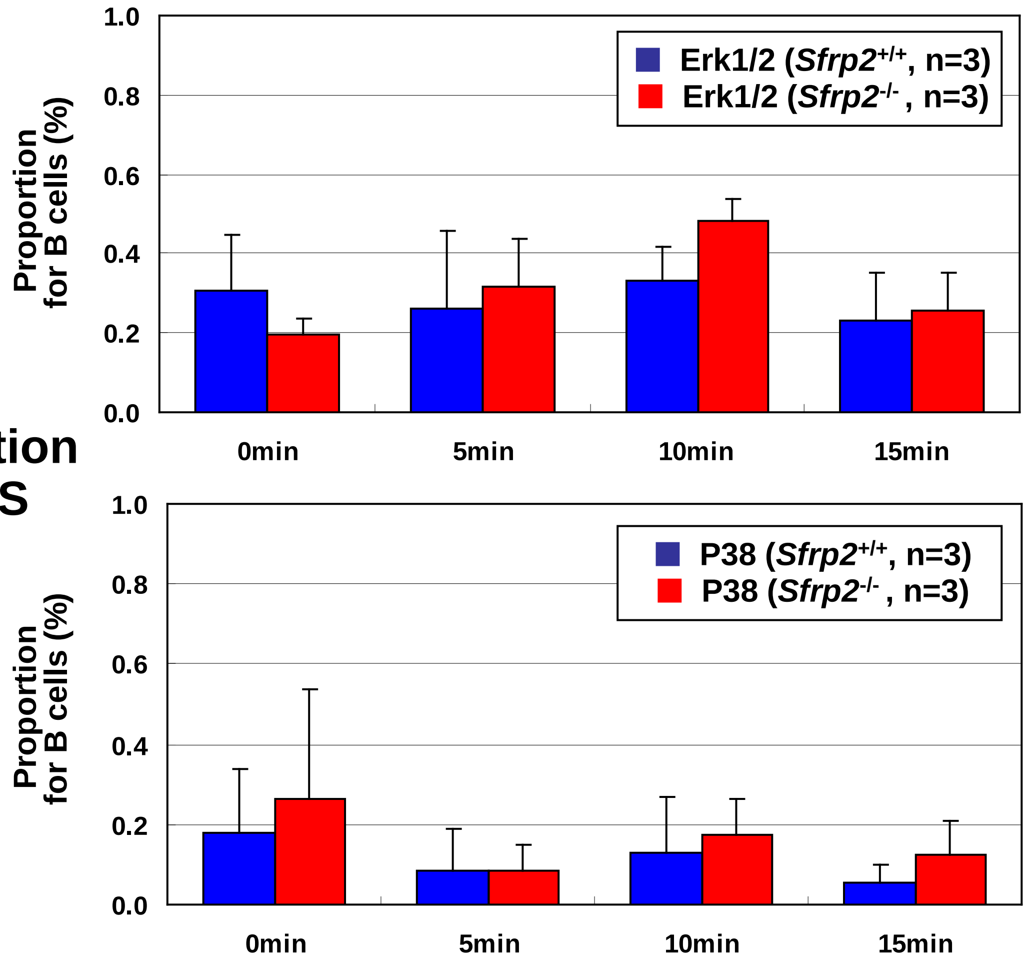

(A) The results of the protein phosphorylations in splenic B cells is demonstrated representatively. The assay was performed by FACS with BD™ Phosflow (BD Biosciences) in time course of 0, 5, 10, and 15 min. The phosphorylations of each protein with the stimulation by IgM were detected by antibodies of Erk1/2 and Syk. In the case of the stimulation by LPS, the phosphorylations were similarly detected by Erk1/2 and P38 antibodies. The histogram shows the distributions according to the intensity of antibody signal.

(B) The histograms in the case of the stimulation by IgM are demonstrated with means and SDs (n=3). There are no significant differences between *Sfrp2*$^{+/+}$ and *Sfrp2*$^{-/-}$ splenic B cells in each time with either *t*-test or Wilcoxon rank sum test.

(C) The histograms in the case of the stimulation by LPS are displayed. There are no significant differences between *Sfrp2*$^{+/+}$ and *Sfrp2*$^{-/-}$ in each time with either *t*-test or Wilcoxon rank sum test.
